# Supplementary material for: Prediction model and technical and tactical decision analysis of women’s badminton singles based on machine learning
Source: PLoS One. 2024 Nov 14;19(11):e0312801. doi: 10.1371/journal.pone.0312801 (PMC11563442; doi:10.1371/journal.pone.0312801)
Supplement: Attachment — Submitted filename: Response to the reviewer 9,7.docx [file pone.0312801.s003.docx]

**Response to the comments**

Dear reviewers:

Data source:

The videos used in this study are available on a variety of video websites, but the official BWF website is limited by copyright issues, and the same competition videos and data are available on the same resources on the YouTube video sharing platform. I have described the source of data in the research methodology section of the manuscript: the badminton competition videos used were sourced from the YouTube video sharing platform, which is a video resource that can be used for public scientific research (for non-profit purposes).

Regarding Youtube's use of copyrighted resources, this URL for copyrighted use is as follows:https://support.google.com/youtube/answer/9783148?hl=en
